# Observatório AlenRiscos - Prevention of the consumption of psychoactive substances among adolescents 3^rd^ cycle

**DOI:** 10.1590/0034-7167-2024-0432

**Published:** 2025-07-11

**Authors:** Ana Maria Guégués da Silva Dias, Ermelinda do Carmo Valente Caldeira, Leonel Lusquinhos de Sousa Oliveira, Lara Manuela Guedes de Pinho, Jorge Manuel Azevedo dos Santos, Maria Margarida Palma Goes, Anabela Pereira Coelho, Manuel José Lopes

**Affiliations:** I Universidade de Évora. Évora, Portugal; II Universidade de Évora, Comprehensive Health Research Centre. Évora, Portugal; III Escola Superior de Enfermagem de Lisboa, Nursing Research Innovation and Development Centre of Lisbon. Lisboa. Portugal

**Keywords:** Students, Psychoactive Substances, Prevention and Control, Schools, Health., Estudiantes, Sustancias Psicoativas, Prevención y Control, Escuelas, Salud.

## Abstract

**Objectives:**

to assess the prevalence and factors associated with psychoactive substance consumption among 7^th^ and 9^th^ grade students.

**Methods:**

an exploratory observational study, through the application of an online self-completion questionnaire, from 2016 to 2022, to 12,767 students.

**Results:**

tobacco, alcohol and drug consumption fluctuated, with alcohol being the most consumed substance by young people, followed by drugs and, lastly, tobacco. The study revealed a downward trend in tobacco and alcohol consumption among adolescents. There was a notable increase in glue, varnish and solvent consumption, which surpassed consumption cannabis/hashish consumption.

**Conclusions:**

initial substance consumption occurs predominantly in a social context and begins in early adolescence. The propensity to consume increases as students age, with higher consumption being noted among older adolescents.

## RESUMEN

**Objetivos:**

evaluar la prevalencia y factores asociados al consumo de sustancias psicoactivas entre estudiantes de 7º y 9º año de escolaridad.

**Métodos:**

estudio observacional exploratorio, mediante la aplicación de un cuestionario de autocumplimentación en línea, durante el período de 2016 a 2022, para 12.767 estudiantes.

**Resultados:**

el consumo de tabaco, alcohol y drogas fluctuó, siendo el alcohol la sustancia más consumida por los jóvenes, seguido de las drogas y, finalmente, el tabaco. El estudio reveló una tendencia a la baja en el consumo de tabaco y alcohol entre los adolescentes. Existe un consumo notoriamente elevado de pegamentos, barnices y disolventes, superando al consumo de cannabis/hachís.

**Conclusiones:**

el consumo inicial de sustancias ocurre predominantemente en un contexto social, y comienza en una fase prematura de la adolescencia. La propensión al consumo aumenta a medida que los estudiantes envejecen, notándose un mayor consumo entre los adolescentes de mayor edad.

## INTRODUCTION

The emergence of psychoactive substance consumption, including tobacco and alcohol, at increasingly early ages is one of the most significant public health challenges on a global scale. According to the World Health Organization (WHO), it is estimated that around 10% of the world’s urban population is involved in the abusive use of these substances, with alcohol, tobacco and cannabis/hashish standing out due to their high prevalence among young people^(1)^.

Given the complexity of this challenge, health observatories play an indispensable role, functioning as platforms for monitoring and analyzing public health trends.

In the particular scenario of Alentejo, *Observatório AlenRiscos* emerges as an innovative initiative, the result of a synergy between the academic community and civil society, aiming to establish a solid basis for an effective intervention adapted to local specificities. The collaboration between experts from the *Universidade de Évora* and professionals from key institutions, such as Évora Addictive Behaviors and Dependencies Intervention Division (In Portuguese, *Divisão de Intervenção dos Comportamentos Aditivos e das Dependências* - DICAD) of *Administração Regional de Saúde do Alentejo* (ARS Alentejo), reinforces a collaborative and multidisciplinary approach in the study of risk behaviours.

Since its inception with the “Know Global, Act Local” project in 2013, the observatory has evolved to adopt a continuous observational methodology, aiming to provide insights that facilitate more targeted and informed interventions by local decision-makers, healthcare services and educational institutions.

Thus, *Observatório AlenRiscos* is established as a strategic response to the needs for intervention in the region, through systematic data collection and analysis. It constitutes a key initiative for the development of more focused and effective educational and public health interventions, aligned with the objectives of conducting regional diagnostic assessments, generating empirical knowledge, identifying new patterns of consumption and addictions, and promoting evidence-based intervention strategies.

The concept of a health observatory, which focuses on monitoring health patterns in a specific population, originated in France in 1974 and quickly spread throughout the health sector at regional level. This methodology was gradually adopted by several European nations and, in the 1990s, became a fundamental pillar of public health in the United Kingdom^(2)^. The organizational setup of health observatories has been recognized globally for its invaluable capacity to foster knowledge sharing and strategic guidance. In 2016, the health observatory model was recognized as essential for population health surveillance, emerging as the preferred structure for detailed monitoring of emerging trends^(3)^. These entities are tasked with providing relevant, high-quality health information to a broad spectrum of users, including healthcare professionals, managers and decision-makers, ensuring access to reliable data and enabling rapid responses to regional health challenges^(2)^.

A thorough understanding of the profile of psychoactive substance use in a specific cohort is a cornerstone for designing targeted and effective interventions. A systematic assessment methodology is a crucial indicator for measuring the impact of intervention measures implemented over a given period^(4)^.

In the context of the problem under analysis, international literature reflects an increasingly complex reality, marked by increased accessibility and greater diversification in the patterns of psychoactive substance consumption^(5)^. At the same time, there is a trend towards starting to use these substances at increasingly younger ages. As reported by the European School Survey Project on Alcohol and Other Drugs (ESPAD), in 2020, more than 18% of students assessed in various European countries reported having started using tobacco at the age of 13 or younger. Percentages vary significantly between countries, with Latvia (31%) and Lithuania (33%) standing out as examples where this phenomenon is particularly pronounced.

As revealed by ESPAD, alcohol is the most commonly consumed substance among adolescents in Europe, with a prevalence of 79%, with the highest rates in Hungary, Denmark and the Czech Republic. Tobacco ranks second, with an average consumption of 41%, with variations ranging from 15% in Iceland to 58% in Slovakia. Cannabis/hashish accounts for the third highest consumption rate among the substances assessed, with 16%. In terms of illicit drug consumption, the average indicates that 19% of boys and 14% of girls reported having consumed these substances at least once in their lives^(6)^.

In Portugal, a study conducted in 2019 by the Service for Intervention in Addictive Behaviors and Dependencies (In Portuguese, *Serviço de Intervenção nos Comportamentos Aditivos e nas Dependências* - SICAD) on alcohol, tobacco, drug consumption and other addictive behaviors and dependencies among students aged 13 to 18, at a national level, revealed that alcohol is the substance predominantly consumed by this young segment, and is also perceived as the most accessible^(7)^. Tobacco consumption is the second most commonly used substance, with an upward trend in the use of electronic cigarettes, in contrast to a decrease or stabilization in conventional cigarette consumption. As regards illicit drugs, cannabis/hashish stands out as the most prevalent among young people in this age group. However, the results indicate a slight reduction or stabilization in the prevalence of consumption of these substances, compared to the data from the previous study^(7)^.

Young people between the ages of 13 and 18 exhibit particular characteristics specific to this period of development that increase their susceptibility to risky behaviors, adversely affecting their physical, mental, psychological, moral and/or social health. Of particular note is the propensity to consume psychoactive substances and the negative impacts resulting from these practices^(8,9)^. These behaviors constitute a serious public health problem in adolescents, due to the long-term consequences that can arise. The use of psychoactive substances by adolescents is associated with reduced academic performance as well as changes in the development of cognitive-behavioral and emotional skills^(10,11)^. The combination of psychoactive substances, including alcohol and other drugs, can trigger severe, immediate or long-term consequences, some of which are potentially lethal. Harmful practices among adolescents are linked to a significant increase in morbidity and mortality in adulthood. It is worth noting that early initiation of this consumption exponentially increases the risk of serious adverse outcomes in the future^(12,13)^.

The WHO defines adolescence as the period between 10 and 19 years of age, a stage of development characterized by a series of physical, mental and social changes that culminate in the distinctive characteristics of adulthood. During this period, the social influence exerted by the group of friends or peers, together with factors such as family environment and school context, can significantly influence harmful substance consumption^(14)^. The use of psychoactive substances, such as tobacco, alcohol and other drugs, by adolescents often serves as a mechanism to reinforce their popularity and status within the peer group, and is often seen as a means of affirming their popularity and position within their social circle^(11,15)^.

Several studies highlight the importance of focusing prevention efforts and promoting healthy lifestyles at early stages of development, specifically between the ages of 11 and 14^(16)^. This approach is based on the understanding that awareness of risks is intrinsically associated with a decrease in the likelihood of initiating the consumption of these substances^(17)^.

This highlights the relevance of studying this issue and the need for concerted involvement by the entire educational community and strategic partners in the areas of health, education, research and social intervention. This study presents the results regarding alcohol, tobacco and other drug consumption by 7^th^ and 9^th^ grade students, both in school clusters and in non-clustered educational establishments in the Alentejo region.

Thus, this study aimed to answer the following question: what is the prevalence and factors associated with psychoactive substance consumption among 7^th^ and 9^th^ grade students?

## OBJECTIVES

To assess the levels of alcohol, tobacco and other psychoactive substance consumption among 7^th^ and 9^th^ grade students in the Alentejo region and analyze the factors associated with them.

## METHODS

### Ethical aspects

In accordance with the Declaration of Helsinki on Ethics in research involving human beings, this study was conducted in accordance with national and international ethics guidelines, subject to prior authorization from the Directorate-General for Education and approval from the management bodies of the school groups involved.

The data collection instrument required written informed consent from all parents or guardians of participants, ensuring total confidentiality and anonymity of the information collected, which is intended exclusively for the purposes of this research.

### Study design, period and location

This study is exploratory and observational, with a method structured through the STrengthening the Reporting of OBservational studies in Epidemiology checklist, using data contained in the observatory’s database, covering the Alentejo region in the time interval from 2016 to 2022. The data contained in the observatory’s database were collected through the annual application of a questionnaire, thus facilitating the monitoring of adolescents’ health behaviors in relation to harmful substance consumption.

### Population or sample; inclusion and exclusion criteria

The study universe included adolescent students, from both public and private schools, enrolled in the 3^rd^ cycle of primary education (corresponding to the 7^th^ and 9^th^ grades of education), in the Alentejo region. This group was selected after obtaining informed consent from their respective guardians, totaling 1,2767 students.

It was decided to exclude the 2020/2021 academic year, given the COVID-19 pandemic, which made it impossible to apply the planned questionnaire.

### Study protocol

For data collection, an online self-completion questionnaire developed and validated by Carvalho in 1990 was used through the LimeSurvey platform, made available by the *Universidade de Évora*. Applying this questionnaire aimed to determine the prevalence of alcoholic beverages, tobacco and illicit drug consumption as well as to identify correlations between certain social and behavioural determinants and reported consumption. The analytical strategy adopted, extending over a period of five years, allowed each cohort to be assessed using the same data collection instrument twice throughout their school career, specifically in the 7^th^ and 9^th^ grades.

### Analysis of results, and statistics

The data collected were organized in a database in a Microsoft Excel for Windows^®^ spreadsheet and analyzed in Statistical Package for the Social Sciences^®^ version 22, using descriptive statistical analysis, relating to the prevalence of illicit substance consumption, and inferential statistical analysis, in order to identify factors that interfere with this consumption.

## RESULTS

### Sample characterization

A total of 1,2767 students participated in this study over the five academic years, with the lowest participation observed in the 2016-17 academic year (n=2039) and the highest participation in the 2019-20 academic year (n=3023). Of the total participants, 48% were male and 52% were female, in the 7^th^ (n=6332) and 9^th^ (n=6435) grade of education, aged between 11 and 18 years.

### Legal and illegal substance consumption in Alentejo

**Figure 1:**
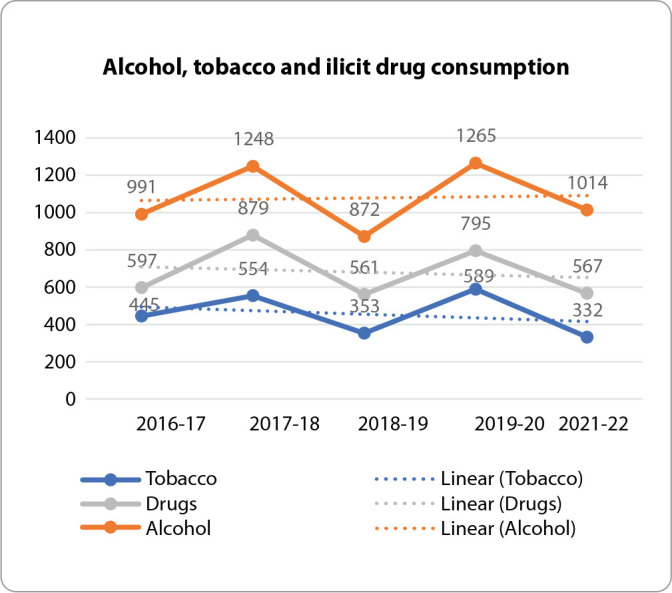
Alcohol, tobacco and illicit drug consumption by school year

Over the five school years of observation, tobacco, alcohol and drug consumption among young people in Alentejo fluctuated, with alcohol being the substance most consumed by young people, followed by drugs and, lastly, tobacco, in all school years (Figure 1).

The school year in which alcohol and tobacco consumption occurred most was 2019-20 (n=1,265 and n=589, respectively). With regard to drugs, consumption was higher in the 2017-18 school year (n=879), as shown in Figure 1.

In the 2021-22 academic year, the greatest decrease in drug and tobacco consumption occurred, with 567 and 332 students, respectively (Figure 1).

### Tobacco

Regarding tobacco consumption, the majority of students reported that they had never smoked in their lives in all academic years, with 2021-22 being the year in which 87.8% of students reported never having smoked.

Students who reported smoking at least once or more in their lives, in the 2016-17 year, corresponded to 21.8%, having decreased to 12.2% in the 2021-22 academic year.

Most participants in this study reported that, when they started smoking, it was in the company of friends, 75.7% in the 2016-17 school year and 65.4% in the 2021-22 school year, and then, alone, 16.2% in 2016-17, having increased to 24.4% in the 2021-22 school year, with this option of starting consumption being the one that increased the most.

Table 1 presents, for the 95% Confidence Intervals, the regression parameters, which, in the case of the 7^th^ and 9^th^ grade school year, are -0.460 and -1.256, respectively, significantly different from 0. Thus, it can be stated that tobacco consumption decreases significantly throughout the school years, both in the 7^th^ and 9^th^ grades.

**Table 1:** Lifetime tobacco use by year of education

Coefficients^a^									
	**Model**		**Unstandardized coefficients**		**Standardized coefficients**	**T**	**Sig.**	**95% Confidence Interval for B**	
			**B**	**Standard error**	**Beta**			**Lower Bound**	**Upper Bound**
**7^th^GRADE**	1	(Constant)	901	90		10.1	0.000%	725	1076
		School year	-0.460	0.045	-0.130	-10.3	0.000%	-0.547	-0.373
		Age	2.252	0.086	0.332	26.3	0.000%	2.084	2.419
**9^th^ GRADE**	1	(Constant)	2480	191		13.0	0.000%	2105	2855
		School year	-1.256	0.095	-0.170	-13.2	0.000%	-1.443	-1.069
		Age	4.056	0.186	0.281	21.8	0.000%	3.691	4.421

*Note: a. Dependent variable: lifetime tobacco use.*

Similarly, it can be seen that age has the effect of significantly increasing tobacco consumption, as its coefficient is 2.252 for the 7^th^ grade and 4.056 for the 9^th^ grade (Table 1).

### Alcohol

Regarding alcohol consumption, the number of students who have never tried alcohol in their lives has increased over the academic years, from 51.4% in 2016-17 to 62.8% in 2021-22. Students who have consumed alcohol once or more have seen the opposite trend, i.e., from 48.6% in 2016-17 to 37.2% in 2021-22.

When study participants were asked about who they started drinking alcohol with, it was consistent across all school years that friends were the people with whom young people first consumed alcohol, followed by family, with family being the only one that increased in the 2021-22 school year (42.9%).

With regard to alcohol consumption, Table 2 presents the 95% Confidence Intervals for the regression parameters, which, in the case of the 7^th^ and 9^th^ grade school year, are -0.702 and -1.284, respectively, significantly different from 0. Thus, it can be stated that alcohol consumption decreases significantly throughout the 7^th^ and 9^th^ grade school year.

**Table 2:** Lifetime alcohol consumption by year of education

Coefficients^a^									
	**Model**		**Unstandardized coefficients**		**Standardized coefficients**	**T**	**Sig.**	**95% Confidence Interval for B**	
			**B**	**Standard error**	**Beta**			**Lower Bound**	**Upper Bound**
**7^th^GRADE**	1	(Constant)	1382	109		12.7	0.000%	1169	1595
		School year	-0.702	0.054	-0.162	-13.0	0.000%	-0.808	-0.596
		Age	2.999	0.104	0.361	28.9	0.000%	2.795	3.203
**9^th^ GRADE**	1	(Constant)	2535	233		10.9	0.000%	2079	2991
		School year	-1.284	0.116	-0.144	-11.1	0.000%	-1.511	-1.057
		Age	4.582	0.226	0.263	20.2	0.000%	4.138	5.026

*Note: a. Dependent variable: lifetime tobacco use.*

Similarly, it can be seen that age has the effect of increasing alcohol consumption due to its coefficient of 2.999 in the 7^th^ grade and 4.582 in the 9^th^ grade (Table 2).

### Illicit drugs

Regarding drug use, it was found that the substance least used by the largest number of participants was crack, throughout all the school years under study, and the most consumed were glues, varnishes and solvents in all school years, although with a downward trend, as shown in Table 3.

**Table 3:** Number of times you have used drugs in life per school year

		School year				
		**2016-17** **n (%)**	**2017-18** **n (%)**	**2018-19** **n (%)**	**2019-20** **n (%)**	**2021-22** **n (%)**
Cannabis/hashish/weed	0	1922 (94.3%)	2705 (94.1%)	2007 (95.5%)	2815 (93.1%)	2640 (96.8%)
	1 or more	117 (5.7%)	171 (5.9%)	95 (4.5%)	208 (6.9%)	87 (3.2%)
Glues, varnishes and solvents	0	1522 (74.6%)	2121 (73.7%)	1613 (76.7%)	2370 (78.4%)	2239 (82.1%)
	1 or more	**517 (25.4%)**	**612 (26.3%)**	**489 (23.3%)**	**653 (21.6%)**	**488 (17.9%)**
Tranquilizers, sedatives^*^	0	2004 (98.3%)	2816 (97.9%)	2072 (98.6%)	2965 (98.1%)	2680 (98.3%)
	1 or more	35 (1.7%)	60 (2.1%)	30 (1.4%)	58 (1.9%)	**47 (1.7%)**
Stimulants, amphetamines	0	2012 (98.7%)	2837 (98.6%)	2087 (99.3%)	2981 (98.6%)	2696 (98.9%)
	1 or more	27 (1.3%)	39 (1.4%)	15 (0.7%)	42 (1.4%)	31 (1.1%)
LSD and other hallucinogens	0	2018 (99.0%)	2844 (98.9%)	2085 (99.2%)	2982 (98.6%)	2704 (99.2%)
	1 or more	21 (1.0%)	32 (1.1%)	17 (0.8%)	41 (1.4%)	23 (0.8%)
Crack	0	**2024 (99.3%)**	**2847 (99.0%)**	**2087 (99.3%)**	**2990 (98.9%)**	**2706 (99.2%)**
	1 or more	15 (0.7%)	29 (1.0%)	15 (0.7%)	33 (1.1%)	21 (0.8%)
Cocaine	0	2015 (98.8%)	2817 (97.9%)	2080 (99.0%)	2979 (98.5%)	2691 (98.7%)
	1 or more	24 (1.2%)	59 (2.1%)	22 (1.0%)	44 (1.5%)	36 (1.3%)
Heroin	0	2023 (99.2%)	2833 (98.5%)	2086 (99.2%)	2991 (98.9%)	2705 (99.2%)
	1 or more	16 (0.8%)	43 (1.5%)	16 (0.8%)	32 (1.1%)	22 (0.8%)
Ecstasy	0	2021 (99.1%)	2842 (98.8%)	2088 (99.3%)	2987 (98.8%)	2702 (99.1%)
	1 or more	18 (0.9%)	34 (1.2%)	14 (0.7%)	36 (1.2%)	25 (0.9%)
Inhalants	0	2013 (98.7%)	2835 (98.6%)	2088 (99.3%)	2989 (98.9%)	2699 (99.0%)
	1 or more	26 (1.3%)	41 (1.4%)	14 (0.7%)	34 (1.1%)	28 (1.0%)
Total		2039 (100.0%)	2876 (100.0%)	2102 (100.0%)	3023 (100.0%)	2727 (100.0%)

Note:

* without medical indication.

In relation to drug use, Table 4 presents the 95% Confidence Intervals for regression parameters, which, in the case of the 7^th^ and 9^th^ grade school year, are -0.676 and -1.18, respectively, significantly different from 0. Thus, it can be stated that drug use decreases significantly throughout the 7^th^ and 9^th^ grade school year.

**Table 4:** Lifetime drug use by year of education

Coefficients^a^									
	**Model**		**Unstandardized coefficients**		**Standardized coefficients**	**T**	**Sig.**	**95% Confidence Interval for B**	
			**B**	**Standard error**	**Beta**			**Lower Bound**	**Upper Bound**
**7^th^GRADE**	1	(Constant)	1331	491		2.7	0.676%	368	2294
		School year	-0.676	.244	-.037	-2.8	0.570%	-1.155	-.197
		Age	3.01	.469	.085	6.4	0.000%	2.092	3.931
**9^th^ GRADE**	1	(Constant)	2344	570		4.1	0.004%	1226	3462
		School year	-1.18	.284	-.055	-4.2	0.003%	-1.737	-.624
		Age	3.10	.554	.075	5.6	0.000%	2.015	4.189

*Note: a. Dependent variable: lifetime tobacco use.*

It is also found that age has the effect of increasing drug consumption, since its coefficient is 3.01 in the 7^th^ grade and 3.10 in the 9^th^ grade. Thus, it can be stated that drug consumption increases significantly with the increase in students’ age (Table 4).

## DISCUSSION

This study found a global trend of reduction in tobacco use among students in Alentejo in line with global trends highlighted by the Global Youth Tobacco Surveys. This latest report points to a generalized decrease in the prevalence of tobacco use among adolescents in 143 countries from 1999 to 2018^(18)^. Despite annual fluctuations, including an increase from 16.8% to 19.5% between the 2018/2019 and 2019/2020 school years, the long-term trajectory revealed a marked reduction, reaching the lowest consumption rate recorded, 12.2%, in the 2021/2022 school year. Additionally, the prevalence of tobacco use, in a period of at least one day in the last 30 days, was 17-9% among boys and 11-5% among girls^(18)^, reiterating the critical importance of tobacco control strategies targeting adolescents globally.

A longitudinal study involving 303 adolescents, aged between 14 and 18 years, carried out before and two months after the confinement imposed by the COVID-19 pandemic, revealed a downward trend in substance use during this period^(19)^. At the same time, in this study, the analysis carried out six months after the end of lockdown supported this downward trend. This phenomenon can be attributed to the change in the social context of adolescents who, during lockdown, were predominantly under the supervision of their parents, restricting the opportunities for substance consumption usually associated with socializing with friends. This context suggests that the dynamics of social interaction play a fundamental role in young people’s consumption behavior, highlighting the need to consider these factors when designing prevention and intervention strategies.

In parallel with the reduction in tobacco consumption, a study covering 87,532 Korean adolescents, conducted to examine fluctuations in alcohol consumption between 2019 and 2021, revealed a downward trend in consumption during 2020 and 2021 compared to 2019^(20)^. This finding is paralleled by the results obtained in this study for the Alentejo region, where there was a decrease in the number of students who reported consuming alcohol in the 2021/2022 school year compared to previous years. It was also observed that, in most cases, the onset of alcohol consumption among adolescents occurred in social contexts with friends, in line with what is documented in scientific literature, which identifies the social influence exerted by peers as a preponderant factor for problematic alcohol consumption among young people^(21)^. A careful review of existing studies indicates that the choice of friends and social interactions are strongly associated with alcohol and tobacco consumption, with this association being particularly pronounced with regard to alcohol^(22)^.

The 2019 ESPAD report identifies cannabis/hashish as the most widely consumed illicit drug in Europe, with an average consumption rate of 16%, while Portugal records a slightly lower rate (13%)^(20)^. In comparison, other substance consumption, such as glues, varnishes and solvents, has an average of 7.2% in Europe and 4.5% in Portugal, showing different consumption patterns between substances and geographical contexts. Additionally, the 2022 Health Behaviour in School-aged Children (HBSC/WHO) study, conducted by the WHO, reiterates that, excluding alcohol and tobacco, cannabis/hashish continues to be the most used substance by adolescents, with a prevalence of 4%, followed by the use of glues, varnishes and other solvents, with 3.7%^(23)^.

The comparative analysis between consumption patterns identified by the 2022 HBSC/WHO and the results obtained in this study reveals notable discrepancies regarding the use of specific substances. While HBSC/WHO points to glue, varnish and other solvent consumption in Europe of 7.2%, and in Portugal, of 4.5%, this study observes significantly higher rates in Alentejo, with percentages ranging from 17.9% to 26.3% in the academic years analyzed. Additionally, cannabis/hashish, although it is the most consumed illicit substance according to the HBSC/WHO report, with 4% prevalence, followed by glues, varnishes and others, with 3.7%, contrasts with regional data, where glue, varnish and solvent consumption exceeds that of cannabis/hashish, which presented rates between 3.2% and 6.9%. As highlighted by Johnston *et al*. (2022), there was stability in glue, varnish and other solvent consumption until 2021, at which point a general decline in the use of these substances among young people in various age groups was observed. This downward trend is echoed in the results of this study, which documents a reduction in the consumption of these substances from 26.3% in 2017 to 17.9% in 2021, suggesting a change in drug use patterns among young people in Alentejo^(24)^.

These results highlight the importance of considering regional and cultural specificities when analysing substance use behaviours among adolescents. Furthermore, they highlight the need to develop intervention strategies tailored to the local context, capable of effectively intervening in youth consumption trends, especially in regions where use patterns diverge significantly from the national or European average. Furthermore, when reflecting on the dynamics of substance use among young people, it is imperative to acknowledge the limitations inherent to the present study. Specifically, the generalizability of the findings is restricted by the peculiarities of the regional context analysed and by interruption in data collection during the 2020/2021 academic year, a direct consequence of the challenges imposed by the COVID-19 pandemic. This time gap highlights the importance of continuous and adaptive studies that can provide more comprehensive information on consumption trends in times of global crisis as well as in normative contexts.

### Study limitations

This study presents as limitations the impossibility of generalizing the results to other geographical and cultural contexts and the interruption in data collection during the 2020/2021 academic year due to the pandemic, recognizing the need for continuity and expansion of research in this area.

### Contributions to nursing, health, or public policy

The results of this study are of considerable relevance for health and nursing practice, as they may provide a solid basis for the construction of effective strategies adapted to this population. They also contribute to the formulation of health policies capable of responding to the challenges identified. This study allows the training of nurses in the area of health promotion regarding harmful substance consumption by adolescents.

## CONCLUSIONS

The data reveal that initial substance use occurs predominantly in a social context, especially among friends, and begins in early adolescence. The propensity to use increases as students age, with higher consumption being noted among older adolescents. However, the period examined shows a downward trend in consumption, particularly marked in the 2021/2022 school year, after the pandemic. This variation suggests a possible influence of the longer period of family life during confinement, which may have reduced opportunities for group consumption.

These results highlight the critical importance of an early and integrated intervention approach that involves not only educational and healthcare settings, but also the family nucleus and the wider community. The implementation of intervention policies and practices should therefore be informed by a deep understanding of the pathways of substance use and the social factors that influence addictive behaviours, thus promoting an effective and sustained response that contributes to mitigating associated risks and fostering the well-being and comprehensive health of adolescents.

The role played by *Observatório AlenRiscos* is of critical importance in this context, by providing detailed, current and highly relevant information that serves as a foundation for the formulation of pragmatic and effective strategies in the face of this evolving phenomenon.

Future studies should seek to explore not only the continuity of observed trends, but also the effectiveness of implemented interventions, adjusting them as necessary to maximize their impact on preventing substance use among young people.
