# Supplementary material for: Observatório AlenRiscos - Prevention of the consumption of psychoactive substances among adolescents 3rd cycle
Source: Rev Bras Enferm. 2025 Jul 11;78(3):e20240432. doi: 10.1590/0034-7167-2024-0432 (PMC12252923; doi:10.1590/0034-7167-2024-0432)
Supplement: Translation

# Translation: Observatório AlenRiscos - Prevenção do consumo de substâncias psicoativas entre adolescentes do ensino básico

**DOI:** 10.1590/0034-7167-2024-0432pt

**Authors:** Ana Maria Guégués da Silva Dias, Ermelinda do Carmo Valente Caldeira, Leonel Lusquinhos de Sousa Oliveira, Lara Manuela Guedes de Pinho, Jorge Manuel Azevedo dos Santos, Maria Margarida Palma Goes, Anabela Pereira Coelho, Manuel José Lopes

**Affiliations:** I Universidade de Évora. Évora, Portugal; II Universidade de Évora, Comprehensive Health Research Centre. Évora, Portugal; III Escola Superior de Enfermagem de Lisboa, Nursing Research Innovation and Development Centre of Lisbon. Lisboa. Portugal

**Keywords:** Estudantes, Substâncias Psicoativas, Prevenção e Controle, Escolas, Saúde.

## Abstract

**Objetivos:**

avaliar a prevalência e os fatores associados ao consumo de substâncias psicoativas entre estudantes do 7º e 9º ano de escolaridade.

**Métodos:**

estudo exploratório observacional, através da aplicação de um questionário de autopreenchimento *online*, durante o periodo de 2016 a 2022, para 12.767 estudantes.

**Resultados:**

o consumo relativo ao tabaco, álcool e drogas sofreu oscilações, sendo o álcool a substância mais consumida pelos jovens, seguida das drogas e, por último, o tabaco. O estudo revelou uma tendência decrescente no consumo de tabaco e álcool entre os adolescentes. Regista-se, de forma notória, um consumo substancialmente elevado de colas, vernizes e solventes, ultrapassando o consumo de *cannabis*/haxixe.

**Conclusões:**

o consumo inicial de substâncias ocorre predominantemente em contexto social, e inicia-se em uma fase prematura da adolescência. A propensão ao consumo aumenta com o avançar da idade dos estudantes, sendo notório um consumo mais elevado entre os adolescentes mais velhos.

## INTRODUÇÃO

A emergência do consumo de substâncias psicoativas, incluindo o tabaco e o álcool, em idades cada vez mais precoces, configura-se como um dos mais significativos desafios de saúde pública à escala global. Segundo a Organização Mundial da Saúde (OMS), estima-se que cerca de 10% da população urbana mundial esteja implicada no uso abusivo dessas substâncias, destacando-se o álcool, o tabaco e a *cannabis/*haxixe, pela sua prevalência acentuada entre os joven^(1)^.

Perante a complexidade deste desafio, os observatórios de saúde assumem um papel indispensável, funcionando como plataformas para a monitorização e análise das tendências de saúde pública.

No cenário particular do Alentejo, emerge o Observatório AlenRiscos como uma iniciativa inovadora, resultado de uma sinergia entre a comunidade académica e a sociedade civil, visando estabelecer uma base sólida para uma intervenção eficaz e adaptada às especificidades locais. A colaboração entre especialistas da Universidade de Évora e profissionais de instituições-chave, como a Divisão de Intervenção dos Comportamentos Aditivos e das Dependências (DICAD) de Évora da Administração Regional de Saúde do Alentejo (ARS Alentejo), reforça uma abordagem colaborativa e multidisciplinar no estudo dos comportamentos de risco.

Desde a sua conceção com o projeto “Conhecer Global, Atuar Local” em 2013, o observatório evoluiu para adotar uma metodologia observacional contínua, visando proporcionar *insights* que facilitem intervenções mais direcionadas e fundamentadas pelos decisores locais, serviços de saúde e instituições educativas.

Assim, o Observatório AlenRiscos estabelece-se como uma resposta estratégica às necessidades de intervenção na região, através da recolha e análise sistemática de dados. Constitui-se como uma iniciativa primordial para o desenvolvimento de intervenções educativas e de saúde pública mais enfocadas e eficazes, alinhadas com os objetivos de conduzir avaliações diagnósticas regionais, gerar conhecimento empírico, identificar novos padrões de consumo e dependências, e promover estratégias de intervenção baseadas em evidências.

A génese do conceito de observatório de saúde, centrado na monitorização de padrões de saúde em uma população específica, teve lugar na França, em 1974, e rapidamente se alastrou pelo setor da saúde a nível regional. Esta metodologia foi progressivamente adotada por várias nações europeias e, na década de 1990, converteu-se em um pilar fundamental da saúde pública no Reino Unido^(2)^. A configuração organizacional dos observatórios de saúde tem sido reconhecida globalmente pela sua capacidade inestimável de fomentar a partilha de conhecimento e orientações estratégicas. Em 2016, o modelo de observatório de saúde foi consagrado como essencial para a vigilância da saúde da população, emergindo como a estrutura preferencial para o acompanhamento detalhado de tendências emergentes^(3)^. Tais entidades são encarregues de fornecer informação de saúde relevante e de alta qualidade a um espectro abrangente de utilizadores, incluindo profissionais de saúde, gestores e decisores, garantindo o acesso a dados confiáveis e possibilitando respostas rápidas a desafios regionais de saúde^(2)^.

A compreensão aprofundada do perfil de consumo de substâncias psicoativas em coorte específica se revela uma pedra angular para a conceção de intervenções direcionadas e eficazes. Uma metodologia de avaliação sistemática se assume como um indicador crucial para mensurar o impacto das medidas de intervenção implementadas em um determinado período^(4)^.

No contexto da problemática em análise, a literatura internacional reflete uma realidade crescentemente complexa, marcada por uma acessibilidade ampliada e por uma maior diversificação nos padrões de consumo de substâncias psicoativas^(5)^. Paralelamente, observa-se uma tendência de iniciação ao consumo dessas substâncias em idades cada vez mais jovens. Conforme reportado pelo *European School Survey Project on Alcohol and Other Drugs* (ESPAD), em 2020, mais de 18% dos estudantes avaliados nos diversos países da Europa relatam ter iniciado o consumo de tabaco aos 13 anos de idade ou menos. As percentagens variam, significativamente, entre países, destacando-se a Letónia (31 %) e a Lituânia (33%) como exemplos onde este fenómeno é particularmente pronunciado.

Conforme revelado pelo ESPAD, o álcool surge como a substância predominantemente consumida pelos adolescentes na Europa, com uma prevalência de 79%, destacando-se as taxas mais altas na Hungria, Dinamarca e República Checa. O tabaco ocupa a segunda posição, com uma média de consumo de 41%, observando-se variações desde 15%, na Islândia, até 58%, na Eslováquia. A *cannabis*/haxixe representa o terceiro maior índice de consumo entre as substâncias avaliadas, com 16%. Em termos de consumo de drogas ilícitas, a média indica que 19% dos rapazes e 14% das raparigas reportaram ter consumido essas substâncias pelo menos uma vez na vida^(6)^.

Em Portugal, o estudo conduzido em 2019 pelo Serviço de Intervenção nos Comportamentos Aditivos e nas Dependências (SICAD) sobre o consumo de álcool, tabaco, drogas e outros comportamentos aditivos e dependências entre os estudantes dos 13 aos 18 anos, a nível nacional, revelou que o álcool é a substância predominantemente consumida por este segmento jovem, sendo também percecionada como a mais acessível^(7)^. Em sequência, o consumo de tabaco surge como a segunda substância mais utilizada, notando-se uma tendência ascendente no uso de cigarros eletrónicos, em contraste com uma diminuição ou estabilização no consumo dos cigarros convencionais. No que concerne a drogas ilícitas, a *cannabis*/haxixe distingue-se como a mais prevalente entre os jovens nesta faixa etária. Todavia, os resultados apontam para uma ligeira redução ou estabilização na prevalência do consumo dessas substâncias, comparativamente aos dados do estudo anterior^(7)^.

Os jovens entre 13 e 18 anos exibem particularidades próprias desse período de desenvolvimento que potenciam a suscetibilidade a comportamentos de risco, afetando adversamente a sua saúde física, mental, psicológica, moral e/ou social. Destaca-se, em especial, a propensão para o consumo de substâncias psicoativas e para os impactos negativos decorrentes dessas práticas^(8,9)^. Esses comportamentos constituem um grave problema de saúde pública nos adolescentes, devido às consequências a longo prazo que podem advir. O consumo de substâncias psicoativas por adolescentes se associa a um desempenho escolar reduzido, bem como a alterações no desenvolvimento de competências cognitivo-comportamentais e emocionais^(10,11)^. A combinação de substâncias psicoativas, incluindo álcool e outras drogas, pode desencadear consequências severas, imediatas ou de longo prazo, algumas das quais potencialmente letais. As práticas nocivas à saúde entre os adolescentes estão vinculadas a um aumento significativo da morbimortalidade na idade adulta. Salienta-se que a iniciação precoce nesse consumo eleva exponencialmente o risco de surgirem resultados adversos graves no futuro^(12,13)^.

A OMS delimita a adolescência como o intervalo dos 10 aos 19 anos, uma etapa de desenvolvimento caracterizada por uma série de mudanças, físicas, mentais e sociais, que culminarão com as características distintivas da idade adulta. Nesse período, a influência social exercida pelo grupo de amigos ou pares, em conjunto com fatores como o ambiente familiar e o contexto escolar, pode influenciar significativamente o consumo de substâncias nocivas^(14)^. O recurso a substâncias psicoativas, tais como tabaco, álcool e outras drogas, pelos adolescentes, frequentemente, serve como mecanismo de reforço da sua popularidade e estatuto dentro do grupo de pares, e é frequentemente encarado como um meio de afirmação da sua popularidade e posição dentro do seu círculo social^(11,15)^.

Vários estudos sublinham a importância de centrar esforços de prevenção e promoção de estilos de vida saudáveis em fases precoces de desenvolvimento, especificamente entre os 11 e os 14 anos^(16)^. Essa abordagem é fundamentada pelo entendimento de que a consciencialização acerca dos riscos está intrinsecamente associada a uma diminuição da probabilidade de iniciação ao consumo dessas substâncias^(17)^.

Deste modo, evidenciam-se a relevância do estudo dessa problemática e a necessidade de um envolvimento concertado de toda a comunidade educativa e de parceiros estratégicos nos domínios da saúde, educação, investigação e intervenção social. Neste estudo, divulgam-se os resultados relativos ao consumo de álcool, tabaco e outras drogas por parte dos estudantes do 7º e 9º ano de escolaridade, tanto em agrupamentos de escolas quanto em estabelecimentos de ensino não agrupados da região do Alentejo.

Assim, pretende-se, com este estudo, dar resposta à seguinte questão: qual a prevalência e os fatores associados ao consumo de substâncias psicoativas entre estudantes do 7º e 9º ano de escolaridade?

## OBJETIVO

Avaliar os níveis de consumo de álcool, tabaco e outras substâncias psicoativas entre os estudantes do 7º e 9º ano na região do Alentejo e analisar os fatores a eles associados.

## MÉTODOS

### Aspectos éticos

Conforme a Declaração de Helsínquia de Ética em pesquisa envolvendo seres humanos, este estudo foi conduzido de acordo com as diretrizes de ética nacionais e internacionais, condicionado à autorização prévia da Direção-Geral da Educação e à aprovação dos órgãos de gestão dos agrupamentos de escolas envolvidos.

O instrumento de recolha de dados exigiu a obtenção de consentimento informado por escrito de todos os pais ou encarregados de educação dos participantes, garantindo a total confidencialidade e anonimato das informações recolhidas, as quais se destinam exclusivamente aos objetivos desta investigação.

### Desenho, período e local do estudo

O estudo foi do tipo exploratório observacional, com método estruturado através do *checklist STrengthening the Reporting of OBservational studies in Epidemiology*, recorrendo aos dados constantes na base de dados do observatório, abrangendo a região do Alentejo no intervalo temporal de 2016 a 2022. Os dados constantes da base de dados do observatório foram recolhidos através da aplicação anual de um questionário, facilitando, assim, a monitorização dos comportamentos de saúde dos adolescentes em relação ao consumo de substâncias nocivas.

### População ou amostra; critérios de inclusão e exclusão

O universo do estudo englobou estudantes adolescentes, tanto do ensino público quanto do ensino privado, inseridos no 3º ciclo do ensino básico (correspondente aos 7º e 9º anos de escolaridade), na região do Alentejo. Este grupo foi selecionado após obtenção do consentimento esclarecido dos respetivos encarregados de educação, totalizando 1.2767 estudantes.

Optou-se por excluir o ano letivo 2020/2021, atendendo à pandemia de COVID-19, que inviabilizou a aplicação do questionário previsto.

### Protocolo do estudo

Para a recolha de dados, utilizou-se um questionário de autopreenchimento *online*, desenvolvido e validado por Carvalho em 1990, através da plataforma *LimeSurvey*, disponibilizado pela Universidade de Évora. O objetivo da aplicação deste questionário reside na determinação da prevalência do consumo de bebidas alcoólicas, tabaco e drogas ilícitas, bem como na identificação de correlações entre certos determinantes sociais, comportamentais e o consumo referido. A estratégia analítica adotada, estendendo-se por um período de cinco anos, possibilitou que cada coorte fosse avaliada mediante o mesmo instrumento de recolha de dados por duas vezes ao longo do seu percurso escolar, especificamente no 7º e no 9º ano.

### Análise dos resultados e estatística

Os dados coletados foram organizados em banco de dados em planilha do *software Microsoft Excel for Windows^®^* e analisados no programa *Statistical Package for the Social Sciences* ^®^, versão 22, com recurso à análise estatítica descritiva, relativa à prevalência do consumo de substâncias ilicitas, e análise estatítica inferencial, de forma a identificar fatores que interferem nesse consumo.

## RESULTADOS

### Caracterização da amostra

Neste estudo, participaram um total de 1.2767 estudantes ao longo dos cinco anos letivos, tendo-se observado, no ano letivo 2016-17, a menor adesão (n=2039), e no ano letivo 2019-20, a maior adesão (n=3023). Do total dos participantes, 48% eram do sexo masculino, e 52%, do sexo feminino, do 7º (n=6332) e 9º (n=6435) ano de escolaridade, com idades compreendidas entre 11 e 18 anos.

### Consumo de substâncias lícitas e ilícitas no Alentejo

Ao longo dos cinco anos letivos de observação, o consumo relativo ao tabaco, álcool e drogas dos jovens do Alentejo sofreu oscilações, sendo o álcool a substância mais consumida pelos jovens, seguida das drogas, e, por último, do tabaco, em todos os anos letivos (Figura 1).

**Figura 1:** Consumo de álcool, tabaco e drogas ilícitas por ano letivo

O ano letivo no qual ocorreu maior consumo de álcool e de tabaco foi em 2019-20 (n=1.265 e n=589, respetivamente). No que se refere às drogas, o consumo foi superior no ano letivo 2017-18 (n=879), conforme demonstra o Figura 1.

No ano letivo 2021-22, ocorreu a maior diminuição do consumo de drogas e tabaco, com 567 e 332 estudantes, respetivamente (Figura 1).

### Tabaco

No que se refere ao consumo de tabaco, a maioria dos estudantes referiu que nunca fumou na vida em todos os anos letivos, com 2021-22 sendo o ano em que 87,8% dos estudantes afirmaram nunca terem fumado.

Os estudantes que referiram fumar pelo menos uma ou mais vezes na vida, no ano 2016-17, correspondiam a 21,8%, tendo diminuído para 12,2% no ano letivo 2021-22.

A maioria dos participantes neste estudo referiram que, quando começaram a fumar, foi na companhia dos amigos, 75,7% no ano letivo 2016-17 e 65,4% no ano letivo 2021-22, e em seguida, sozinhos, 16,2% em 2016-17, tendo aumentado para 24,4% no ano letivo 2021-22, sendo esta opção de início de consumo a que mais aumentou.

Na Tabela 1, apresentam-se, para os Intervalos de Confiança de 95%, os parâmetros da regressão que, no caso do ano letivo do 7º e 9º ano de escolaridade, são de -0,460 e -1,256, respetivamente, significativamente diferentes de 0. Assim, pode afirmar-se que o consumo de tabaco diminui significativamente ao longo dos anos letivos, tanto no 7º quanto no 9º ano.

**Tabela 1:** Consumo de tabaco ao longo da vida por ano de escolaridade

Coeficientes^a^									
	**Modelo**		**Coeficientes não padronizados**		**Coeficientes padronizados**	**T**	**Sig.**	**Intervalo de Confiança de 95% para B**	
			**B**	**Erro padrão**	**Beta**			**Limite Inferior**	**Limite Superior**
**7º ANO**	1	(Constante)	901	90		10,1	0,000%	725	1076
		Ano letivo	-0,460	0,045	-0,130	-10,3	0,000%	-0,547	-0,373
		Idade	2,252	0,086	0,332	26,3	0,000%	2,084	2,419
**9º ANO**	1	(Constante)	2480	191		13,0	0,000%	2105	2855
		Ano letivo	-1,256	0,095	-0,170	-13,2	0,000%	-1,443	-1,069
		Idade	4,056	0,186	0,281	21,8	0,000%	3,691	4,421

*Nota: a. Variável dependente: consumo de tabaco ao longo da vida.*

De modo análogo, verifica-se que a idade tem o efeito de aumentar, significativamente, o consumo de tabaco, pois o seu coeficiente é de 2,252, para o 7º ano, e de 4,056, para o 9º ano (Tabela 1).

### Álcool

No que se refere ao consumo de álcool, o número de estudantes que nunca experimentou álcool ao longo da vida tem aumentado, ao longo dos anos letivos, de 51,4%, em 2016-17, para 62,8%, em 2021-22. Os estudantes que consumiram álcool uma ou mais vezes têm tido a evolução inversa; isto é, de 48,6%, no ano de 2016-17, diminuiu para 37,2%, em 2021-22.

Quando os participantes do estudo foram questionados sobre com quem iniciaram o consumo de álcool, foi uniforme, ao longo de todos os anos letivos, que os amigos são aqueles com que os jovens consomem pela primeira vez álcool, seguidos da família, tendo este sido o único que aumentou no ano letivo 2021-22 (42,9%).

No que diz respeito ao consumo de álcool, na Tabela 2, apresentam-se os Intervalos de Confiança de 95% para os parâmetros da regressão que, no caso do ano letivo do 7º e 9º ano de escolaridade, são de -0,702 e -1,284, respetivamente, significativamente diferentes de 0. Assim, pode-se afirmar que o consumo de álcool diminui significativamente ao longo do ano letivo do 7º e 9º ano de escolaridade.

**Tabela 2:** Consumo de álcool ao longo da vida por ano de escolaridade

Coeficientes^a^									
	**Modelo**		**Coeficientes não padronizados**		**Coeficientes padronizados**	**T**	**Sig.**	**Intervalo de Confiança de 95% para B**	
			**B**	**Erro padrão**	**Beta**			**Limite Inferior**	**Limite Superior**
**7º ANO**	1	(Constante)	1382	109		12,7	0,000%	1169	1595
		Ano letivo	-0,702	0,054	-0,162	-13,0	0,000%	-0,808	-0,596
		Idade	2,999	0,104	0,361	28,9	0,000%	2,795	3,203
**9º ANO**	1	(Constante)	2535	233		10,9	0,000%	2079	2991
		Ano letivo	-1,284	0,116	-0,144	-11,1	0,000%	-1,511	-1,057
		Idade	4,582	0,226	0,263	20,2	0,000%	4,138	5,026

*Nota: a. Variável dependente: consumo de tabaco ao longo da vida.*

De modo semelhante, verifica-se que a idade tem o efeito de aumentar o consumo de álcool devido ao seu coeficiente de 2,999, no 7º ano, e de 4,582, no 9º ano (Tabela 2).

### Drogas ilicítas

Quanto ao consumo de drogas, verifica-se que a substância menos experimentada pelo maior número de participantes foi o crack, ao longo de todos os anos letivos, em estudo, e as mais consumidas foram as colas, vernizes e solventes em todos os anos letivos, contudo com tendência a diminuir, como demonstra a Tabela 3.

**Tabela 3:** Número de vezes que consumiu droga na vida por ano letivo

		Ano letivo				
		**2016-17** **n (%)**	**2017-18** **n (%)**	**2018-19** **n (%)**	**2019-20** **n (%)**	**2021-22** **n (%)**
*Cannabis* **/**haxixe/erva	0	1922 (94,3%)	2705 (94,1%)	2007 (95,5%)	2815 (93,1%)	2640 (96,8%)
	1 ou mais	117 (5,7%)	171 (5,9%)	95 (4,5%)	208 (6,9%)	87 (3,2%)
Colas, vernizes e solventes	0	1522 (74,6%)	2121 (73,7%)	1613 (76,7%)	2370 (78,4%)	2239 (82,1%)
	1 ou mais	**517 (25,4%)**	**612 (26,3%)**	**489 (23,3%)**	**653 (21,6%)**	**488 (17,9%)**
Tranquilizantes, “drunfos” ou sedativos^*^	0	2004 (98,3%)	2816 (97,9%)	2072 (98,6%)	2965 (98,1%)	2680 (98,3%)
	1 ou mais	35 (1,7%)	60 (2,1%)	30 (1,4%)	58 (1,9%)	**47 (1,7%)**
Estimulantes, anfetaminas ou **“** *speeds* ”^*^	0	2012 (98,7%)	2837 (98,6%)	2087 (99,3%)	2981 (98,6%)	2696 (98,9%)
	1 ou mais	27 (1,3%)	39 (1,4%)	15 (0,7%)	42 (1,4%)	31 (1,1%)
LSD e outros alucinógenos	0	2018 (99,0%)	2844 (98,9%)	2085 (99,2%)	2982 (98,6%)	2704 (99,2%)
	1 ou mais	21 (1,0%)	32 (1,1%)	17 (0,8%)	41 (1,4%)	23 (0,8%)
Crack	0	**2024 (99,3%)**	**2847 (99,0%)**	**2087 (99,3%)**	**2990 (98,9%)**	**2706 (99,2%)**
	1 ou mais	15 (0,7%)	29 (1,0%)	15 (0,7%)	33 (1,1%)	21 (0,8%)
Cocaína	0	2015 (98,8%)	2817 (97,9%)	2080 (99,0%)	2979 (98,5%)	2691 (98,7%)
	1 ou mais	24 (1,2%)	59 (2,1%)	22 (1,0%)	44 (1,5%)	36 (1,3%)
Heroína	0	2023 (99,2%)	2833 (98,5%)	2086 (99,2%)	2991 (98,9%)	2705 (99,2%)
	1 ou mais	16 (0,8%)	43 (1,5%)	16 (0,8%)	32 (1,1%)	22 (0,8%)
Ecstasy	0	2021 (99,1%)	2842 (98,8%)	2088 (99,3%)	2987 (98,8%)	2702 (99,1%)
	1 ou mais	18 (0,9%)	34 (1,2%)	14 (0,7%)	36 (1,2%)	25 (0.9%)
Inalantes	0	2013 (98,7%)	2835 (98,6%)	2088 (99,3%)	2989 (98,9%)	2699 (99,0%)
	1 ou mais	26 (1,3%)	41 (1,4%)	14 (0,7%)	34 (1,1%)	28 (1,0%)
Total		2039 (100,0%)	2876 (100,0%)	2102 (100,0%)	3023 (100,0%)	2727 (100,0%)

Nota:

* sem indicação médica.

Quanto ao consumo de drogas, na Tabela 4, apresentam-se os Intervalos de Confiança de 95% para os parâmetros da regressão que, no caso do ano letivo do 7º e 9º ano de escolaridade, são de -0,676 e -1,18, respetivamente, significativamente diferentes de 0. Assim, pode-se afirmar que o consumo de drogas diminui significativamente ao longo do ano letivo do 7º e 9º ano.

**Tabela 4:** Consumo de drogas ao longo da vida por ano de escolaridade

Coeficientes^a^									
	**Modelo**		**Coeficientes não padronizados**		**Coeficientes padronizados**	**T**	**Sig.**	**Intervalo de Confiança de 95% para B**	
			**B**	**Erro padrão**	**Beta**			**Limite Inferior**	**Limite Superior**
**7º ANO**	1	(Constante)	1331	491		2,7	0,676%	368	2294
		Ano letivo	-0,676	,244	-,037	-2,8	0,570%	-1,155	-,197
		Idade	3,01	,469	,085	6,4	0,000%	2,092	3,931
**9º ANO**	1	(Constante)	2344	570		4,1	0,004%	1226	3462
		Ano letivo	-1,18	,284	-,055	-4,2	0,003%	-1,737	-,624
		Idade	3,10	,554	,075	5,6	0,000%	2,015	4,189

*Nota: a. Variável dependente: consumo de tabaco ao longo da vida.*

Verifica-se ainda que a idade tem o efeito de aumentar o consumo de drogas, pois o seu coeficiente de 3,01, no 7º ano e de 3,10, no 9º ano. Assim, pode afirmar-se que o consumo de drogas aumenta significativamente com o aumento da idade dos estudantes (Tabela 4).

## DISCUSSÃO

No presente estudo, constatou-se uma tendência global de redução no consumo de tabaco entre os estudantes no Alentejo, alinhando-se com as tendências globais destacadas pelo *Global Youth Tobacco Surveys*. Este último relatório aponta para uma diminuição generalizada na prevalência do consumo de tabaco entre adolescentes em 143 países, de 1999 a 2018^(18)^. Apesar de observadas flutuações anuais, incluindo um incremento de 16,8% para 19,5% entre os anos letivos de 2018/2019 e 2019/2020, a trajetória de longo prazo revelou uma redução acentuada, atingindo a menor taxa de consumo registada, 12,2%, no ano letivo de 2021/2022. Adicionalmente, a prevalência de consumo de tabaco, em um período mínimo de um dia nos últimos 30 dias, foi de 17-9% entre rapazes e 11-5% entre raparigas^(18)^, reiterando a importância crítica de estratégias de controlo do tabaco direcionadas aos adolescentes em âmbito global.

Estudo longitudinal envolvendo 303 adolescentes, com idades entre 14 e 18 anos, realizado antes e dois meses após o confinamento imposto pela pandemia de COVID-19, revelou uma tendência de decréscimo no consumo de substâncias durante esse período^(19)^. Paralelamente, neste estudo, a análise efetuada seis meses após o término do confinamento corroborou essa tendência de redução. Esse fenómeno pode ser atribuído à mudança no contexto social dos adolescentes que, durante o confinamento, encontravam-se predominantemente sob a supervisão dos pais, restringindo as oportunidades para o consumo de substâncias habitualmente associadas à socialização com os amigos. Tal contexto sugere que as dinâmicas de interação social desempenham um papel fundamental no comportamento de consumo dos jovens, apontando para a necessidade de considerar esses fatores na conceção de estratégias de prevenção e intervenção.

Paralelamente à redução no consumo de tabaco, estudo abrangendo 87.532 adolescentes coreanos, realizado para examinar as flutuações no consumo de álcool entre 2019 e 2021, revelou uma tendência de redução no consumo durante 2020 e 2021, comparativamente a 2019^(20)^. Essa descoberta encontra paralelo nos resultados obtidos neste estudo para a região do Alentejo, onde se registou uma queda no número de estudantes que reportaram consumir álcool no ano letivo de 2021/2022 face aos anos precedentes. Observou-se também que, na maioria dos casos, o início do consumo de álcool entre os adolescentes ocorreu em contextos sociais com amigos, indo ao encontro do que é documentado na literatura científica, que identifica a influência social exercida pelos pares como um fator preponderante para o consumo de álcool de forma problemática entre os jovens^(21)^. Revisão criteriosa dos estudos existentes indica que a escolha de amigos e as interações sociais estão fortemente associadas ao consumo de álcool e tabaco, sendo essa associação particularmente mais acentuada no que concerne ao álcool^(22)^.

O relatório de 2019 do ESPAD identifica a *cannabis*/haxixe como a droga ilícita mais consumida na Europa, apresentando uma taxa média de consumo de 16%, enquanto Portugal regista uma taxa ligeiramente inferior (13%)^(20)^. Comparativamente, o consumo de outras substâncias, como colas, vernizes e solventes, apresenta uma média de 7,2% na Europa e de 4,5% em Portugal, evidenciando padrões de consumo diferenciados entre substâncias e contextos geográficos. Adicionalmente, o estudo *Health Behaviour in School-aged Children* (HBSC/OMS), de 2022, conduzido pela OMS, reitera que, excluindo o álcool e o tabaco, a *cannabis*/haxixe continua a ser a substância mais utilizada por adolescentes, com uma prevalência de 4%, seguida pelo uso de colas, vernizes e outros solventes, com 3,7%^(23)^.

A análise comparativa entre os padrões de consumo identificados pelo HBSC/OMS de 2022 e os resultados obtidos neste estudo revela discrepâncias notáveis no que se refere ao uso de substâncias específicas. Enquanto o HBSC/OMS aponta para um consumo na Europa de colas, vernizes e outros solventes de 7,2%, e em Portugal, de 4,5%, este estudo observa taxas significativamente mais elevadas no Alentejo, com percentagens variando de 17,9% a 26,3% nos anos letivos analisados. Adicionalmente, a *cannabis*/haxixe, embora seja a substância ilícita mais consumida segundo o relatório do HBSC/OMS, com 4% de prevalência, seguida por colas, vernizes e outros, com 3,7%, contrasta com os dados regionais, onde o consumo de colas, vernizes e solventes excede o da *cannabis*/haxixe, que apresentou taxas entre 3,2% e 6,9%. Conforme destacado por Johnston *et al*. (2022), houve uma estabilidade no consumo de colas, vernizes ou outros solventes até 2021, momento a partir do qual se observa um declínio geral no uso dessas substâncias entre os jovens em várias faixas etárias. Essa tendência de decréscimo encontra eco nos resultados do presente estudo, que documenta uma redução no consumo dessas substâncias de 26,3%, em 2017, para 17,9%, em 2021, sugerindo uma mudança nos padrões de consumo de drogas entre os jovens no Alentejo^(24)^.

Esses resultados sublinham a importância de atentar às especificidades regionais e culturais na análise dos comportamentos de consumo de substâncias entre adolescentes. Ademais, evidenciam a necessidade de desenvolver estratégias de intervenção moldadas ao contexto local, aptas a intervir de forma eficaz nas tendências de consumo juvenil, especialmente em regiões onde os padrões de uso divergem significativamente da média nacional ou europeia. Além disso, ao refletir sobre as dinâmicas de consumo de substâncias entre os jovens, é imperativo reconhecer as limitações inerentes ao presente estudo. Especificamente, a generalização das conclusões encontra-se restringida pelas peculiaridades do contexto regional analisado e pela interrupção na recolha de dados durante o ano letivo de 2020/2021, uma consequência direta dos desafios impostos pela pandemia de COVID-19. Esta lacuna temporal sublinha a importância de estudos contínuos e adaptativos que possam fornecer informações mais abrangentes sobre as tendências de consumo em períodos de crise global, bem como em contextos normativos.

### Limitações do estudo

Este estudo apresenta como limtações a impossibilidade de generalizar os resultados para outros contextos geográficos e culturais e a interrupção na recolha de dados durante o ano letivo de 2020/2021 devido à pandemia, reconhecendo-se a necessidade de continuidade e expansão da investigação nessa área.

### Contribuições para as áreas da enfermagem, saúde, ou políticas públicas

Os resultados deste estudo são de considerável relevância para a saúde e para a prática de enfermagem, uma vez poderá oferecer uma base sólida para a construção de estratégias eficazes adaptadas a essa população. Também contribui para a formulção de políticas de saúde capazes de responder aos desafios identificados. Este estudo permite a capacitação dos enfermeiros no âmbito da promoção da saúde relativamente ao consumo de substâncias nocivas pelos adolescentes.

## CONCLUSÕES

Os dados revelam que o consumo inicial de substâncias ocorre predominantemente em contexto social, especialmente entre amigos, e inicia-se em uma fase prematura da adolescência. A propensão ao consumo aumenta com o avançar da idade dos estudantes, sendo notório um consumo mais elevado entre os adolescentes mais velhos. No entanto, o período examinado mostra uma tendência de redução no consumo, particularmente acentuada no ano letivo de 2021/2022, após a pandemia. Essa variação sugere uma possível influência do maior período de convivência familiar durante o confinamento, que terá possivelmente reduzido as oportunidades de consumo em grupo.

Esses resultados evidenciam a importância crítica de uma abordagem interventiva precoce e integrada que envolva não só as instâncias educativas e de saúde, mas também o núcleo familiar e a comunidade mais alargada. A implementação de políticas e práticas interventivas deve, portanto, ser informada por um entendimento profundo dos percursos de consumo de substâncias e dos fatores sociais que influenciam os comportamentos aditivos, promovendo, assim, uma resposta eficaz e sustentada que contribua para a mitigação dos riscos associados, e fomentando o bem-estar e a saúde integral dos adolescentes.

O papel desempenhado pelo Observatório AlenRiscos assume uma importância crítica neste contexto, ao disponibilizar informações detalhadas, atuais e de elevada relevância que servem de alicerce para a formulação de estratégias pragmáticas e eficazes face a esse fenómeno em evolução.

Estudos futuros deverão procurar explorar não apenas a continuidade das tendências observadas, mas também a eficácia das intervenções implementadas, ajustando-as conforme necessário para maximizar o seu impacto na prevenção do consumo de substâncias entre os jovens.
